# Design and development of a convolutional neural network based on human cognitive attention mechanism for automatic classification of leukemia

**DOI:** 10.1371/journal.pone.0336770

**Published:** 2026-02-19

**Authors:** Mohammad Zolfaghari, Mohammad Saniee Abadeh, Hedieh Sajedi

**Affiliations:** 1 Faculty of Electrical and Computer Engineering, Tarbiat Modares University, Tehran, Iran; 2 School of Mathematics, Statistics and Computer Science, College of Science, University of Tehran, Tehran, Iran; The University of Alabama in Huntsville, UNITED STATES OF AMERICA

## Abstract

Cancer occurs when healthy cells in the body grow abnormally and out of control. Leukemia is a type of cancer that affects White Blood Cells (WBCs) and can cause a lethal infection and early death. Identification and classification of different types of leukemia are performed manually and automatically. The doctors analyze blood samples under a microscope and consider any changes in the number and structure of WBCs as a sign of cancer in the manual method. It is a time-consuming, inaccuracy-prone process that depends on the expertise and skill of the physician and the type of laboratory equipment. In recent years, more automated methods of identifying and classifying leukemia have been developed with the help of Artificial Intelligence (AI) and Computer Vision (CV), with the aim of overcoming the challenges of manual approaches. This paper introduces two types of attention blocks, Parallel Cognitive Attention Block (PCAB) and Sequential Cognitive Attention Block (SCAB), to integrate into the architecture of any Convolutional Neural Network (CNN). Each of the proposed attention blocks is composed of the channel and spatial attention sub-blocks. They extract the structure and location of WBCs in the feature maps, similar to the ventral and dorsal streams in the human brain. The PCAB and SCAB were embedded in the architecture of the ResNet18 and MobileNetv4. The baseline and attention-based networks are trained, validated, and tested by two types of data splitting on the four leukemia datasets, including ALL, ALL-IDB2, C-NMC, and Mixture-Leukemi (ALL-IDB2+Munich AML Morphology), with the same experimental conditions for 30 epochs. The classification results demonstrate that the proposed model (MobileNetv4PCAB) achieved better performance metrics than others on all datasets in the test steps. It showed that the suggested model achieved the accuracy values of 100%, 100%, 93.61%, and 99.4%, and the F1-score values of 100%, 100%, 95.64%, and 99.3% with ALL, ALL-IDB2, C-NMC, and Mixture-Leukemia datasets, respectively. We confirmed that the proposed model outperforms existing state-of-the-art methods.

## Introduction

White Blood Cells (WBCs) are the main part of the immune system of the human body. They are produced from bone marrow and are generally classified into myeloid and lymphoid cells. Leukemia occurs when abnormal cells of the WBCs proliferate excessively, generated by the bone marrow. Acute Lymphoblastic Leukemia (ALL), Acute Myeloid Leukemia (AML), Chronic Lymphocytic Leukemia (CLL), and Chronic Myeloid Leukemia (CML) are four main kinds of leukemia. ALL mainly affects children, while AML, CLL, and CML are more common in older people [1–4].

Cancer is the second-leading cause of death in the world. Among all forms of cancers, leukemia is 13th in cancer cases and 10th in cancer-associated deaths based on reported information from the International Agency for Research on Cancer (IARC). In the United Kingdom (UK), it is the ninth most commonly detected cancer and the eighth leading cause of cancer-related deaths [5]. Based on the results of the epidemiological trend of leukemia from 1990 to 2021 in 204 countries, it shows about 460,000 cases of leukemia and approximately 320,000 deaths. Population growth has led to an increase in new cases of leukemia in the world, which is very worrying [6]. The US National Cancer Institute’s Surveillance, Epidemiology, and End Results (SEER) database expects 62,770 new cases of leukemia and 23,670 deaths associated with it in the United States of America (USA) in 2024 [7,8]. Such statistics show that leukemia is one of the important cancers, and its timely identification and treatment will prevent the deaths of many affected people.

Manual leukemia identification and classification methods are completely dependent on the science and experience of the hematologists in this field, and the type of medical equipment. They consider the number, morphology, and other related features of the WBCs in this technique. The time and accuracy of leukemia diagnosis are vital for early recognition before development, quick treatment, reducing symptoms and complications, etc. Therefore, techniques based on Deep Neural Networks (DNNs) compared to manual methods offer hopeful avenues to increase the accuracy and efficiency of leukemia detection automatically [9–12].

Convolutional Neural Networks (CNNs) are the most famous and widely used types of DNNs that have been effective in image classification by capturing important features and patterns using a hierarchical architecture of layers. Combining CNNs with different techniques, such as attention blocks, improves their performance metrics. The attention blocks force CNNs to focus on the features related to the Region of Interest (RoI) for more correct decision-making in image classification [13–17].

The biological attention mechanism causes the human brain to process some visual information more quickly and accurately while ignoring other information altogether. Such selective action in the brain reduces memory consumption and information processing time. In image classification problems with CNNs, increasing the number of convolution and pooling layers can improve classification accuracy, but this significantly increases memory consumption, computational load, and model complexity. Also, the risk of gradient vanishing rises by increasing the network depth and may make model training difficult. Moreover, it leads to insufficient generalization ability and increases the probability of overfitting. Designing residual attention blocks inspired by the biological attention mechanism and embedding them in the CNN architecture can improve image classification accuracy in addition to overcoming the mentioned challenges [18–20].

Visual information in the human brain is processed through the ventral and dorsal streams. The ventral pathway extracts the structure of the objects, while the dorsal pathway determines their location. For this reason, these streams are called the ‘what-information’ and ‘where-information’ pathways, respectively. In this study, the Cognitive Attention Blocks (CABs), inspired by the two visual streams in the human brain, in parallel and sequential kinds are designed for embedding in each CNN to aim to enhance leukemia classification. We have designed CABs in two types to comprehensively review their advantages and disadvantages with each other and introduce one of them as a proposed attention block in this paper. Each of the CABs has two sections: the channel and spatial attentions. The channel attention, such as the ventral pathway, emphasizes ‘what’ information by extracting feature maps more relevant to the WBCs structure, while spatial attention, such as the dorsal pathway, processes ‘where’ information by focusing on the location of the WBCs in the selected feature maps.

In this paper, we used four leukemia datasets, including ALL [21], ALL-IDB2 [22], C-NMC [23], and Mixture-Leukemia (ALL-IDB2+Munich AML Morphology [24]) in the experiments. They are different in terms of origin, number of classes, balance and imbalance of class samples, number of WBCs in each image, etc. First, the data augmentation on the ALL-IDB2 dataset, K-fold cross-validation split, and train-validation-test split are performed in the preprocessing step. The original data of the ALL and C-NMC datasets are employed in the experiments. Then, we selected and fine-tuned two CNNs, including the ResNet18 [25] and the MobileNetV4 [26], for implementing on the leukemia datasets. After that, the suggested cognitive attention blocks, including the Parallel Cognitive Attention Block (PCAB) and the Sequential Cognitive Attention Block (SCAB), are designed and embedded in the convolution layers of the selected CNNs. Since convolution layers extract revealing features using combining inter-channel and spatial information, the proposed attention blocks highlight meaningful features along the channel and spatial dimensions. They powerfully aid the information stream inside the network by learning the features associated with the structure and location of the WBCs in the feature maps. The fine-tuned and attention-based networks are trained, validated, and tested on the leukemia datasets with two types of data splitting under the same experimental conditions to determine the role of each proposed attention block in the classification results. Comparing the results of the proposed attention-based model in this study with previous related works on automatic leukemia classification showed that it is superior to them in terms of efficiency and generalizability.

Our primary contributions are as follows:

- Two new CABs in parallel and sequential states are proposed to improve the performance of CNNs for leukemia classification.
- Combining the Global Average Pooling (GAP) and Global Maximum Pooling (GMP) and using the Dilated Convolutional (DC) layer in the proposed attention blocks forces the network to focus more on the structure and position of the WBCs in the feature maps and to increase its accuracy for leukemia classification.
- The effectiveness of the proposed model is proven by comprehensive comparisons of its classification results with previous state-of-the-art methods.

The remainder of the article is organized as follows: the previous state-of-the-art research on the automatic classification of leukemia using microscopic images will be reviewed in the “Related work” section. The section “Materials and methodology” describes the employed datasets, splitting the dataset samples, and the proposed work. Section “Experiment, results, and discussion” explains the experimental setup, hyperparameter tuning, training and test processes, performance metrics, computational complexities, ablation study, comparison with existing models, statistical analysis, visualizing feature maps, and the cases of correct and incorrect predictions. Summarizing the findings of the research and exploring potential avenues for future studies are presented in the “Conclusions and future work” section.

## Related work

The previous studies on automated leukemia classification are collected and arranged according to the approach type and the year of presentation in Table 1. They are categorized into three groups based on approach type: machine learning, deep learning, and hybrid. In the machine learning approach, extracting features is done manually, while classification operations are performed automatically. Feature extraction and classification steps are automatic in the deep learning method. The hybrid method is a combination of machine learning and deep learning approaches. In recent years, researchers in the field of Artificial Intelligence (AI) and Computer Vision (CV) have increasingly used the combination of deep learning networks (especially CNNs) with other techniques to improve automated leukemia classification [27]. Zakir Ullah et al. [28] and Masoudi [29] have used the combination of channel attention mechanism with CNN to improve the network performance measures, while the combination of channel-spatial attention mechanism leads to further improvement of the network in classification. Jawahar et al. [30] introduced a channel-spatial attention block, and only the sequential state of the sub-blocks was investigated, whereas by designing and implementing both sequential and parallel states of the sub-blocks, a more comprehensive and accurate evaluation of them can be achieved. Also, they only used the GAP in the channel attention sub-block in their attention mechanism, while the combination of GAP and GMP would be much more efficient for extracting channels more related to WBCs in leukemia classification.

**Table 1:** Previous studies on automated leukemia classification.

Study	Approach	Year	Dataset	Methodology
Kasani et al. [31]	Deep learning	2020	C-NMC	NasNetLarge and VGG19, Data augmentation, Transfer learning
Praveena and Singh [32]	Hybrid		ALL-IDB2	Segmentation using Sparse Fuzzy C-Means (Sparse FCM) clustering algorithm, Local Directional Patterns (LDP) and Colour Histogram-based features, Customized-CNN and Grey wolf-based Jaya Optimization Algorithm (GreyJOA) classifiers
Sahlol et al. [33]			ALL-IDB2 C-NMC	VGG19 and Statistically Enhanced Salp Swarm Algorithm (SESSA) as the feature selector, Support Vector Machine (SVM) as the classifier
Das and Meher [34]	Deep learning	2021	ALL-IDB2	MobilenetV2 and ResNet18, Transfer learning
Ghaderzadeh et al. [21]			ALL	Segmentation based on color thresholding in the HSV space, DenseNet201, Data augmentation
Zakir Ullah et al. [28]			C-NMC	VGG16, Efficient Channel Attention (ECA), Data augmentation
Jawahar et al. [35]		2022	C-NMC	ALNett, Data augmentation, Transfer learning
Masoudi [29]	Hybrid		ALL-IDB2	HSV color space and EfficientNet-V2M feature extractor, Variable-kernel Channel-spatial Attention (VKCS), Data augmentation
Abhishek et al. [36]		2023	Mixtute	VGG16 features, SVM classifier, Transfer learning
Atteia et al. [37]			ALL	Feature extraction using GoogleNet, Feature selection by hybrid Particle Swarm Optimization (PSO)-Principal Component Analysis (PCA) approach, Bayesian-optimized SVM classifier
Rahman et al. [38]			ALL	ResNet50, Support Vector Classifier (SVC) feature selector, Particle Swarm Optimization (PSO) and Cat Swarm Optimization (CSO), Logistic Regression (LR) classifier
Awais et al. [39]		2024	ALL ALL-IDB2	GoogleNet and customized-CNN, Hybrid Whale optimization (BWO) feature selector, Data augmentation, Transfer learning
Jawahar et al. [30]	Deep learning		ALL	Residual CNN + Channel and Spatial Attention Block (CSAB), Data augmentation, Transfer learning
Shah et al. [40]	Machine learning		C-NMC	Segmentation, Shapes and extracting topological features, XGBoost classifier
Kasim et al. [41]	Hybrid	2025	Mixture (ALL-IDB2+ Munich AML Morphology)	Pre-trained InceptionV3 feature extractor and SVM classifier, Data augmentation
Nagendiran and Murugasamy [42]	Machine learning		ALL ALL-IDB2	Segmentation using Multilevel Hierarchical Marker-Based Watershed Algorithm (MHMW), Morphology, transformation, texture, GLCM, and statistical feature extractors, feature selection by Enhanced Glowworm Swarm Optimization (EGSO), Random Forest (RF) classifier
Shaban [43]			ALL	Leukemia Classification System (LCS), Color segmentation, Morphological and textural features, Feature selection using the Dimensional Archimedes Optimization Algorithm (DAOA), Ensemble Classifier (EC)
Shehta et al. [44]	Deep learning		C-NMC	ResNetRS50, Data augmentation

**Fig 1:**
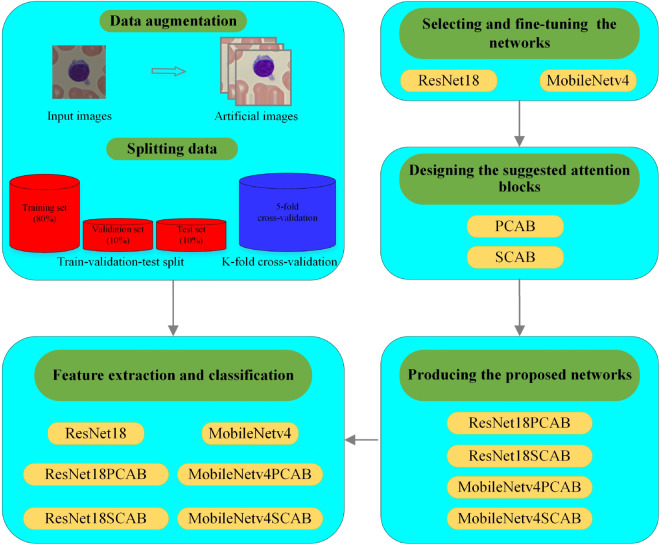
The outline of the proposed methodology.

The graphical representation of the overall proposed methodology in this paper is presented in Fig 1.

## Materials and methodology

We describe the employed datasets, splitting the dataset samples, and the proposed work in this section.

### Employed datasets

**Fig 2:**
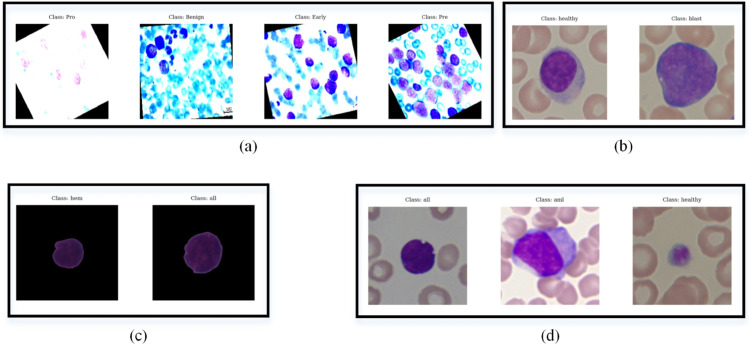
Sample images of the datasets. (a) ALL, (b) ALL-IDB2, (c) C-NMC, and (d) Mixture-Leukemia.

Since classification results on the multiple datasets can justify the effectiveness of the model, four datasets, including ALL, ALL-IDB2, C-NMC, and Mixture-Leukemia, are employed in this work. Table 2 displays the properties of the used datasets. As we can see, they are different in terms of region, the number of samples and classes, image type, and resolution. Sample images of each class of each dataset are shown in Fig 2. As we see, ALL-IDB2 and C-NMC are two-class datasets, while Mixture-Leukemia and ALL are three and four-class datasets. The origin of the ALL and C-NMC is Asian, while ALL-IDB2 and Mixture-Leukemia have European origin. The images of ALL-IDB2, C-NMC, and Mixture-Leukemia datasets have only one WBC, while the ALL dataset includes multiple WBCs in each image. The ALL and ALL-IDB2 have balanced datasets, while the C-NMC and Mixture-Leukemia are unbalanced. Therefore, the networks in this research have been comprehensively trained and evaluated on various leukemia datasets to make the results more reliable and generalizable.

**Table 2:** The properties of the used datasets.

Dataset	Presented by	Year	Samples	Classes	Patients	Image Type	Resolution
ALL	The bone marrow laboratory of Taleqani Hospital, Tehran, Iran	2021	3,256	4	89	Color/JPG	224×224
ALL-IDB2	Department of Computer Science, the University of Milan, Italy	2011	260	2	N/A	Color/TIF	257×257
C-NMC	The Laboratory Oncology Unit, Dr. B.R.A IRCH, All India Institute of Medical Sciences (AIIMS), New Delhi, India	2019	10,661	2	76	Color/BMP	450×450
Munich AML Morphology	The Munich University Hospital, Germany	2019	3,268	1	200	Color/TIFF	400×400

Before implementing the networks on the datasets, we performed data augmentation and split the data in the preprocessing step. Various data augmentation techniques, like as brightness, horizontal and vertical flipping, horizontal and vertical shift (in the ranges of ±0.2 left or right and up or down to half of the actual size of the image), rotation (in the ranges of ±0.45 degrees), scaling (in the ranges of 0.5∼1), and shearing (in the ranges of 0.1∼0.9) are performed on the ALL-IDB2 dataset to increase the samples and reduce the probability of overfitting. We produced the Mixture-Leukemia by combining the ALL-IDB2 and Munich AML morphology datasets.

### Splitting the dataset samples

The samples of all datasets are divided into two different types of data splitting methods: Train-validation-test and K-fold cross-validation splits. These two data splitting methods help to improve the effectiveness, robustness, generalization, and avoid overfitting of the networks. We describe each kind of data splitting in this study in the following.

**Train-validation-test split.** All data are divided into three sections, including train, validation, and test, in this technique. The patterns and relations inside the data are learnt by the network on the training set to make the classification. Hyperparameter tuning and model selection are done using the validation set. The model is evaluated with the simulated real-world scenario by test data. Therefore, preventing overfitting, hyperparameter tuning, and unbiased performance evaluation are the purposes of the train-validation-test split to identify and mitigate overfitting, and to measure the robustness and generalization. We divided 80% of the samples of the ALL, ALL-IDB2, and Mixture-Leukemia datasets for training, 10% for validation, and the rest for testing the networks. Since the images of the C-NMC dataset were in three Folds, the train-validation-test split is performed for each Fold of this dataset. Table 3 shows the splitting of the train-validation-test of data for each dataset.

**Table 3:** Splitting train-validation-test of data.

Dataset		Training	Validation	Test	Total
ALL		2,604	328	324	3,256
ALL-IDB2		800	100	100	1,000
C-NMC	Fold-0	2,822	353	352	3,527
	Fold-1	2,864	359	358	3,581
	Fold-2	2,843	356	354	3,553
Mixture-Leukemia		3,414	427	427	4,268

**Fig 3:**
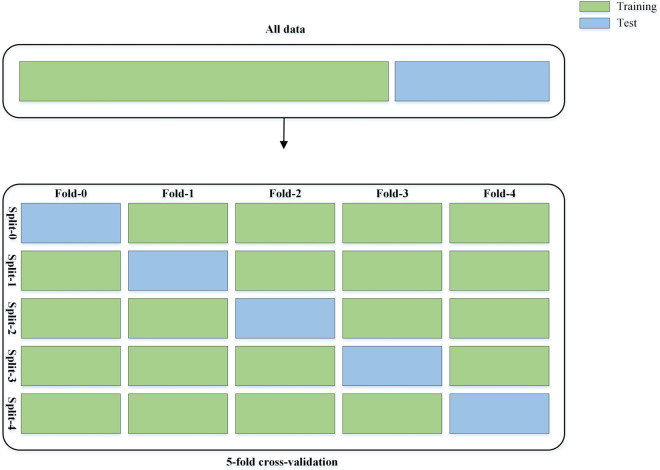
The 5-fold cross-validation procedure.

**K-fold cross-validation split.** In this technique, first, the total samples of each dataset are randomly divided into K equal-sized subsets without overlapping, so that one of the K folds is for the testing set, and the remaining K-1 folds are combined and employed as the training set. Then, this procedure is frequent K times to ensure that each of the K folds is used as the test set exactly once. Finally, while all K iterations are completed, the averages of performance metrics of the folds are calculated [45]. The K-fold cross-validation split is a more comprehensive evaluation compared to the train-validation-test split because each data point in the dataset is used at least once for both the training and test. Also, the variance of the models’ performance criteria is reduced by averaging across the folds in calculating each metric. When we have an imbalanced dataset (like C-NMC and Mixture-Leukemia) where one class is significantly more frequent than the others, the K-fold cross-validation split is usually used to ensure that each fold preserves the same class distribution as the original dataset. We used a 5-fold cross-validation split according to Fig 3 to obtain a comprehensive understanding of its predictive capabilities and generalization ability.

### Proposed work

We employed two CNNs, ResNet18 and MobileNetv4, for embedding the suggested CABs in their architectures in this study. The architecture of each CNN and CAB is explained in detail in the following.

**ResNet18.** The backbone of ResNet18 is composed of a set of Basic Blocks (BBs) that each BB has two direct convolution layers and a residual (shortcut) connection with or without a convolution layer. In the BB0, the residual connection doesn’t have any convolution layer, while BB1 has a 1 × 1 convolution layer. This connection transfers the input with the same dimension in the BB0, while BB1 can convert the input to the desired dimension.

**MobileNetv4.** The latest version of the MobileNets is MobileNetv4, and the Universal Inverted Bottleneck (UIB) search block is in the backbone of it. Extra-DepthWise (Extra-DW), Inverted Bottleneck (IB), and Conv-Next are instantiations of the UIB. Extra-DW combines the benefits of ConvNext-Like and IB, and inexpensively increases the depth and receptive field of the network. Spatial mixing on the extended features’ activations is performed by IB for greater capacity of the network at increased cost. Conv-Next permits cheaper spatial mixing with a larger kernel size by doing the spatial mixing earlier in the expansion.

**Fig 4:**
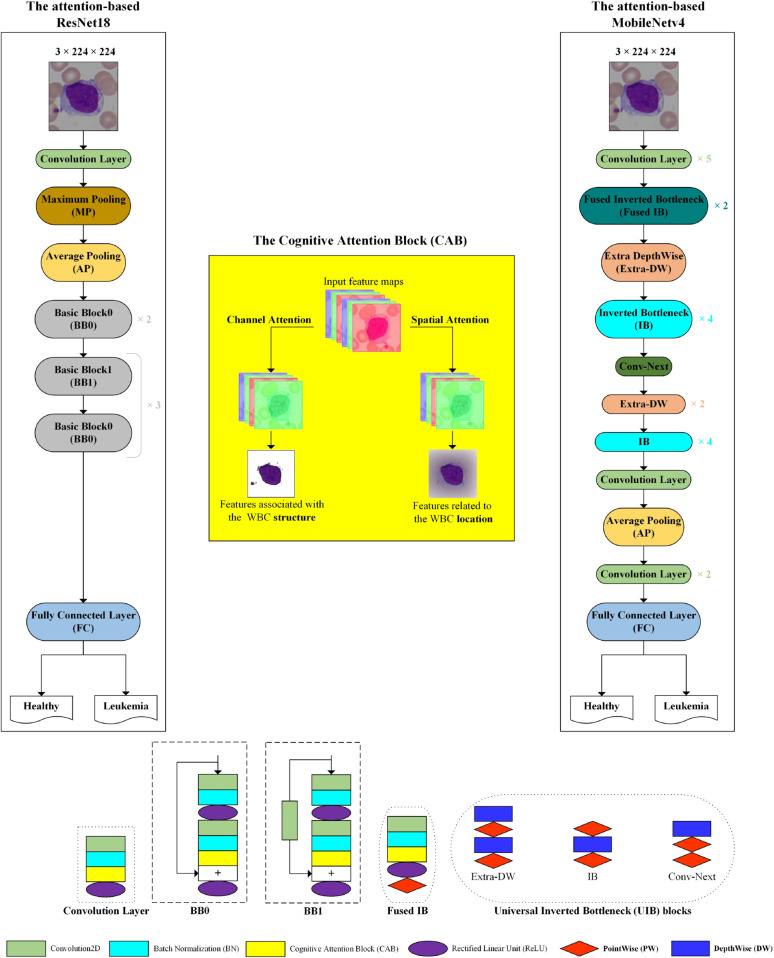
The architecture of the proposed networks.

**Attention-based networks.** When the human focuses on an object in the scene, picture, video, etc, its information are processed through two distinct pathways, the ventral and dorsal, in the human brain based on the Two Visual Systems Hypothesis (TVSH) [46]. The ventral stream extracted the structure of the focused object while the dorsal stream found its location. In other words, they process the ‘What’ and ‘Where’ information from the focused object, respectively. In this study, we designed two channel-spatial attention blocks, PCAB and SCAB, based on the TVSH for increasing the performance metrics of CNNs in automated leukemia classification. They are embedded within the second convolution layers of the BBs in the ResNet18 and all convolution layers of the MobileNetv4. Fig 4 shows the architecture of the proposed networks for automatically classifying microscopic images of leukemia in this study. The dimensions of the images of the datasets are changed to 3×224×224 before commencing training. The ResNet18 and MobileNetv4 are designed for 1,000 classes, and we changed the output neurons of the Fully Connected (FC) layer of them to two, three, and four class classifications. We explain the architecture of the two different types of the proposed CABs, PCAB and SCAB, in detail in the following.

**Fig 5:**
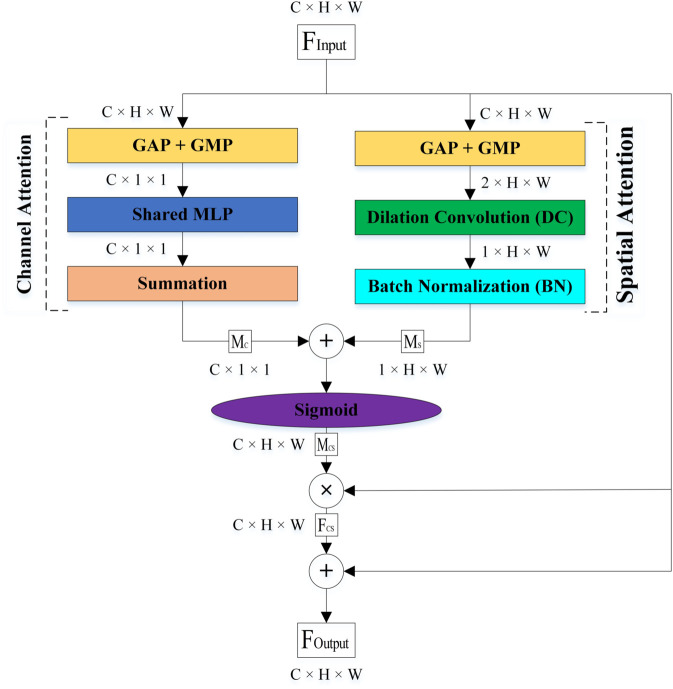
The architecture of the PCAB.

**The PCAB.** The architecture of the PCAB is displayed in Fig 5. It is composed of the channel and spatial attention sub-blocks. They learn the inter-channel and inter-spatial features from the input in parallelly. $F_{Input}$ is the input feature map to the PCAB with C×H×W dimensions. Where C, H, and W indicate the Channels, Height, and Width, respectively. At the beginning of each sub-block, the GAP and GMP are applied on the $F_{Input}$. They compute the average and maximum values of each channel across all spatial positions and focus on its spatial dimensions. The output of the channel attention sub-block is a feature map that only includes channel dimensions related to the structure of WBCs. The output of the spatial attention sub-block is two feature maps with the H×W dimensions, which show the location of the WBCs. The feature maps of the channel and spatial attention sub-blocks pass from a Shared Multi-Layer Perceptron (Shared MLP) and a Dilation Convolution (DC) layer. The Shared MLP has three layers, including an input layer, a hidden layer, and an output layer. The hidden layer is set to $\frac{C}{R}$, where R indicates the reduction ratio. The DC layer effectively expands the kernel size than a standard convolution layer without increasing computational complexity [47]. Thus, the DC layer is used in the spatial attention sub-block instead of the standard convolutional layer. The summation operation and Batch Normalization (BN) are applied on the output feature maps of the Shared MLP and DC layer, respectively, to generate the channel and spatial attention maps ($M_{C}$ and $M_{S}$). They sum together and pass from a sigmoid activation function (σ) to produce the channel-spatial map ($M_{CS}$), and then the output of the channel-spatial attention sub-block ($F_{CS}$) is created by multiplying $F_{Input}$ and $M_{CS}$. Finally, the output feature map of the PCAB ($F_{Output}$) is achieved by the summation of $F_{CS}$ and $F_{Input}$. The overall attention process in the PCAB can be summarized as:

$$F_{Output}=F_{Input}+F_{CS}$$ (1)

$$F_{CS}=F_{Input}⊗M_{CS}$$ (2)

$$M_{CS}=\sigma (M_{C}(F_{Input})+M_{S}(F_{Input}))$$ (3)

$$M_{C}(F_{Input})=MLP(GAP(F_{Input})+GMP(F_{Input}))$$ (4)

$$M_{S}(F_{Input})=BN(DC(GAP(F_{Input})+GMP(F_{Input})))$$ (5)

where + and ⊗ denote element-wise summation and multiplication.

**Fig 6:**
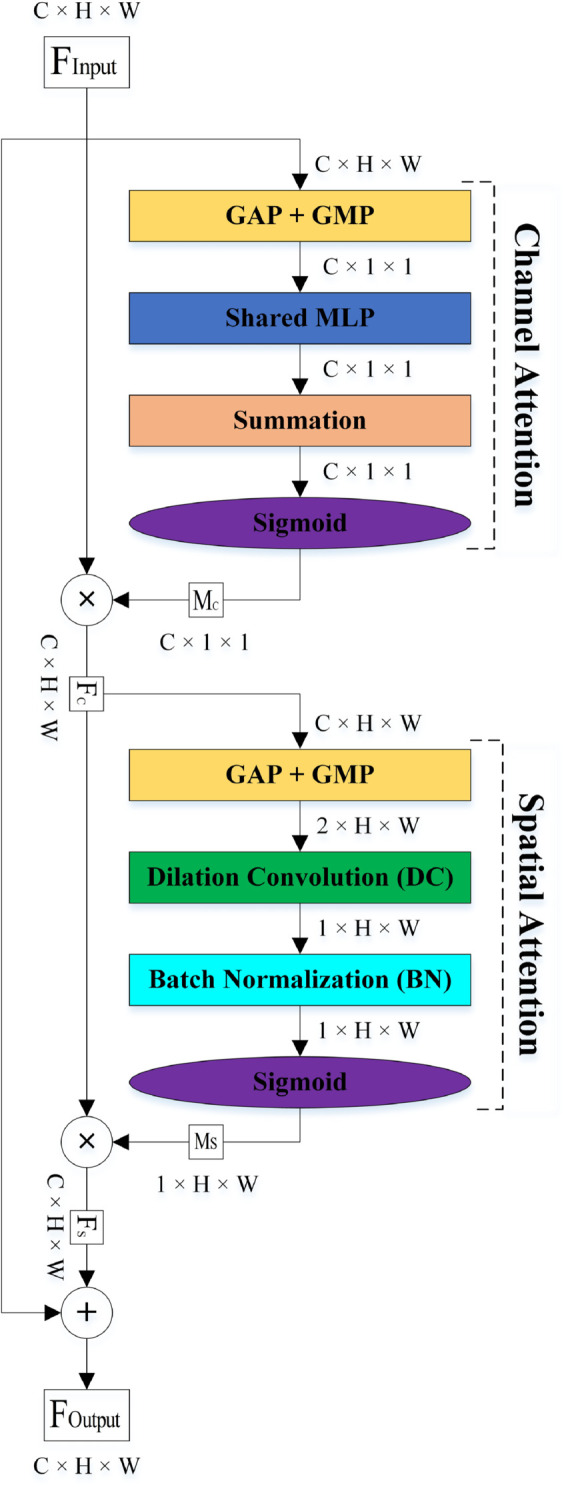
The architecture of the SCAB.

**The SCAB.** The architecture of the SCAB is displayed in Fig 6. In the SCAB, first, the channel attention sub-block finds the inter-channel features, and then the spatial attention sub-block processes the inter-spatial features. After applying the GAP and GMP on the $F_{Input}$ in the channel attention sub-block, a feature map is produced that has only channel dimensions. This feature map is taken to the Shared MLP. The output feature maps of the Shared MLP are summed together and applied a σ for producing the $M_{C}$. The output of the channel attention sub-block ($F_{C}$) is computed by multiplying $M_{C}$ and $F_{Input}$. It is used as the input of the spatial attention sub-block. The GAP and GMP are taken from the $F_{C}$. The output feature maps are passed from the DC and BN layers and σ to generate $M_{S}$. The output of the spatial attention sub-block ($F_{S}$) is produced by multiplying the $M_{S}$ and $F_{C}$. Finally, the output feature map of the SCAB (*F*_*Output*_) is attained by the summation of the *F*_*S*_ and $F_{Input}$. The overall attention process in the SCAB can be summarized as:

$$F_{Output}=F_{Input}+F_{S}$$ (6)

$$F_{S}=F_{C}⊗M_{S}$$ (7)

$$F_{C}=F_{Input}⊗M_{C}$$ (8)

$$M_{C}(F_{Input})=\sigma (MLP(GAP(F_{Input})+GMP(F_{Input})))$$ (9)

$$M_{S}(F_{C})=\sigma (BN(DC(GAP(F_{C})+GMP(F_{C}))))$$ (10)

We embedded and evaluated the proposed attention blocks in different layers of the architecture of the fine-tuned ResNet18 and MobileNetv4. The classification results confirmed that applying them within convolutional layers improves the network’s performance metrics more than in other layers. Therefore, the PCAB and SCAB are separately embedded into the second convolution layers of the BBs in the fine-tuned ResNet18 and all convolutional layers of the fine-tuned MobileNetv4, and four cognitive attention-based networks, including ResNet18PCAB, ResNet18SCAB, MobileNetv4PCAB, and MobileNetv4SCAB, are produced.

## Experiment, results, and discussion

The experimental setup, hyperparameter tuning, training and test processes, performance metrics, computational complexities, ablation study, comparison with existing models, statistical analysis, visualizing feature maps, and the cases of correct and incorrect predictions are explained in this section.

### Experimental setup

Google Colaboratory (Google Colab) is a free and cloud-based platform that permits users to write and execute Python code through the Internet. The proposed methodology is performed using the Graphics Processing Unit (GPU) by the Jupyter Notebook tool. Table 4 shows the experimental hardware and software environments in this study.

**Table 4:** Experimental hardware and software environments.

Hardware/Software	Parameter/Version
Operating System	Windows 11-64 bit
CPU	Intel (R) Core (TM) i7-8565U
GPU	Intel (R) UHD Graphics 620
Python	3.11.12
PyTorch	2.6.0+cu124
Numpy	2.0.2
Opencv	4.11.0

### Hyperparameter tuning

Hyperparameters are external configuration variables that are manually set before training a machine learning model. The performance metrics of each model depended on choosing the correct configuration of hyperparameters. The optimum hyperparameter values were experimentally found by tuning the networks on the datasets in the training step. The networks were trained with the Stochastic Gradient Descent (SGD) optimizer and the Cross-entropy loss function. Table 5 shows the hyperparameter configuration of networks in our experiments.

**Table 5:** Hyperparameter configuration of the networks.

Hyperparameter	Variable	Selected
Batch size	4, 8, 16, 32, 64, 128	16
Dilation ratio	1, 2, 3, 4	2
Epochs	10, 20, 30, 40, 50, 60, 70, 80, 90, 100	30
Learning rate	0.001, 0.002, 0.003, 0.004, 0.005, 0.006, 0.007, 0.008, 0.009	0.001
L2-parameter	0.001, 0.002, 0.003, 0.004, 0.005, 0.006, 0.007, 0.008, 0.009	0.005
Momentum	0.1, 0.2, 0.3, 0.4, 0.5, 0.6, 0.7, 0.8, 0.9	0.9
Reduction ratio	4, 8, 16, 32, 64	16
Weight decay	0.001, 0.002, 0.003, 0.004, 0.005, 0.006, 0.007, 0.008, 0.009	0.005

### Training and test processes

**Fig 7:**
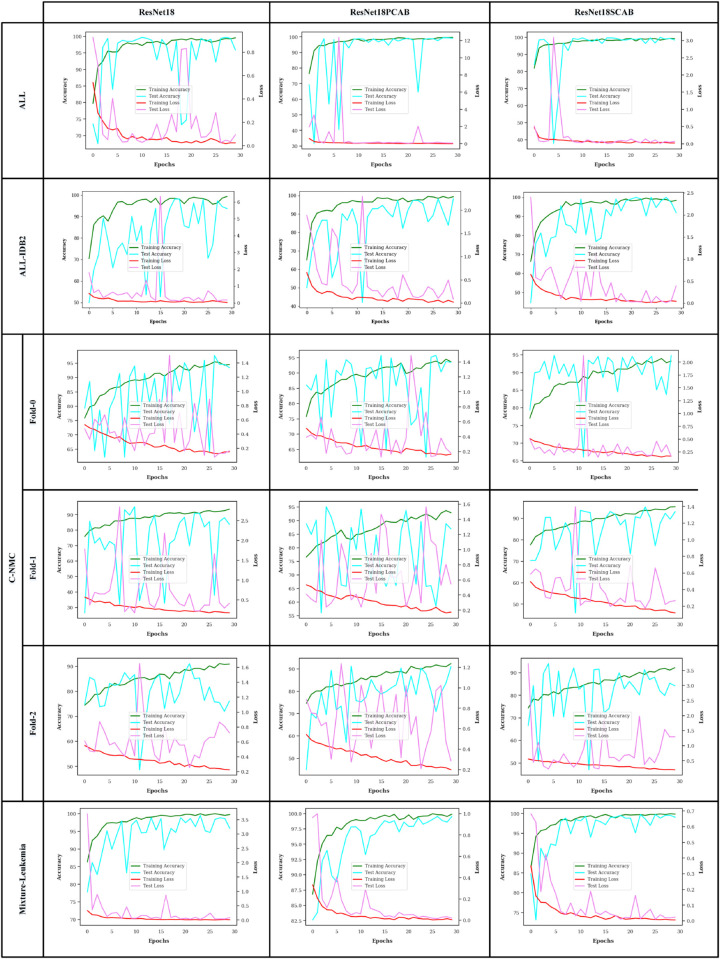
The accuracy and loss curves of the ResNet18 models.

**Fig 8:**
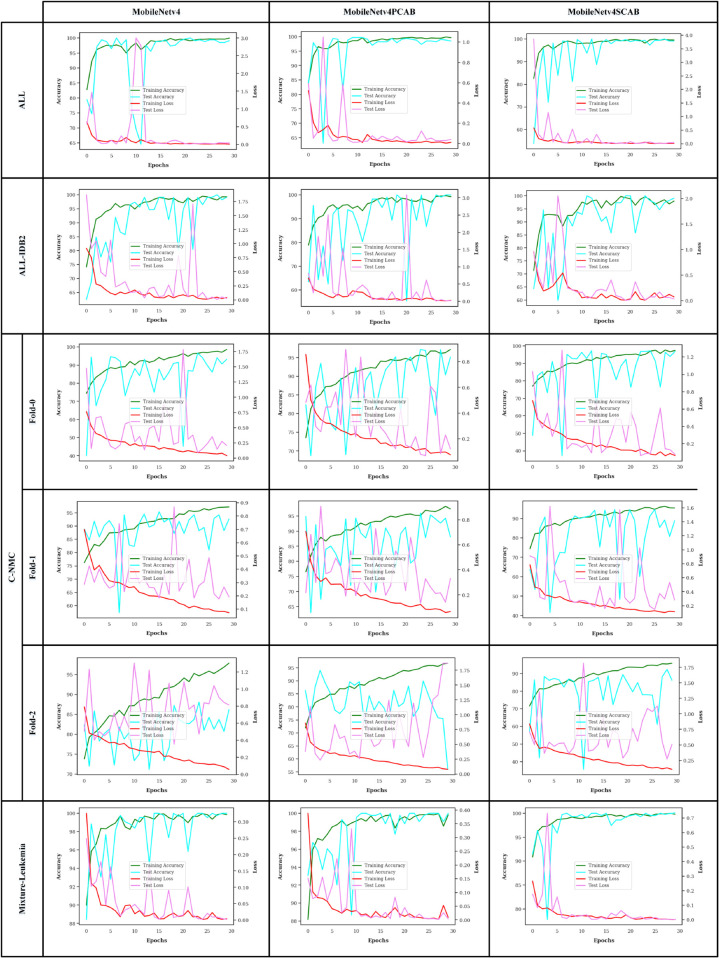
The accuracy and loss curves of the MobileNetv4 models.

Accuracy and loss curves are used to understand how learn well a machine learning model is learning and gets better over time. We display the accuracy and loss curves of the ResNet18 and MobileNetv4 models on the datasets during the training and test steps in Figs 7 and 8, respectively. As we can see, all of the networks reached a good convergence and didn’t experience any overfitting. The attention-based networks, especially the ResNet18SCAB and MobileNetv4SCAB, on all datasets in the training and test phases have had higher accuracy and better convergence than the others.

### Performance metrics

**Fig 9:**
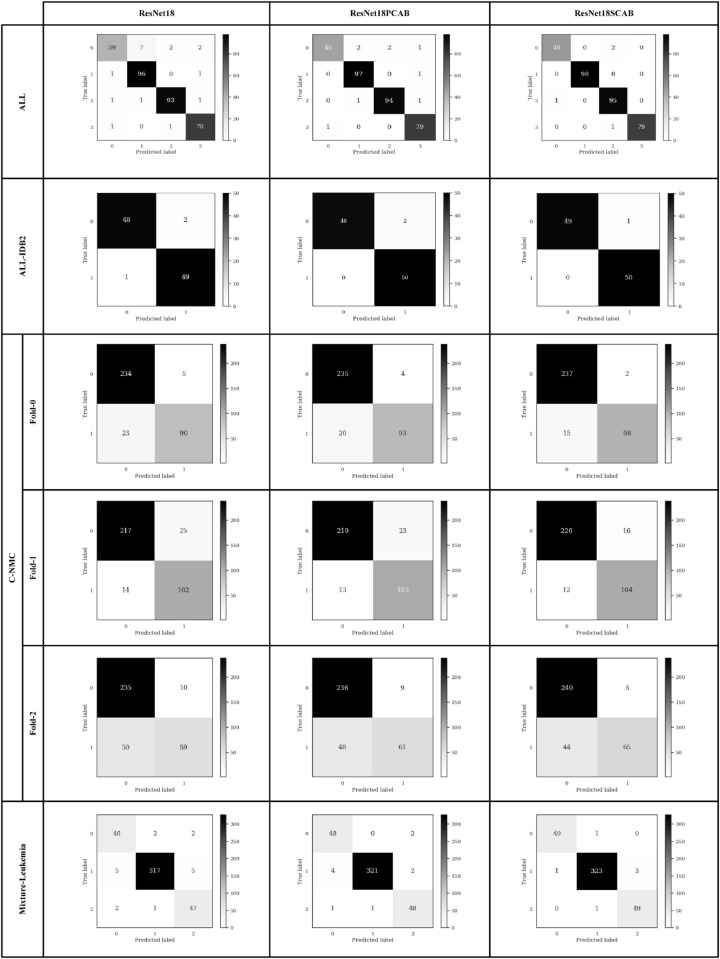
The confusion matrix of the ResNet18 models.

**Fig 10:**
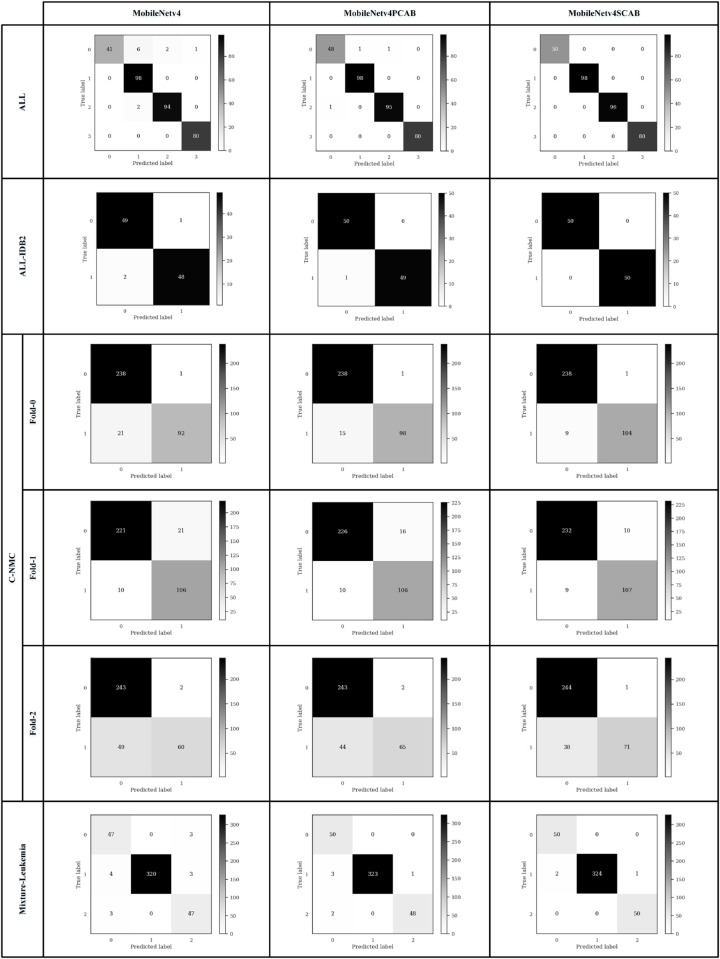
The confusion matrix of the MobileNetv4 models.

The effectiveness of a test or a machine learning model in identifying and classifying leukemia is crucial for correct diagnosis, patient risk assessment, and timely treatment. Performance metrics, including accuracy (Correct Classification Rate (CCR)), precision (Positive Predictive Value (PPV)), sensitivity (recall), specificity, and F1-score relevant to the clinical context of leukemia, quantify the aforementioned tests and models. We used these metrics to evaluate the performance of the networks in the test step. They are the key performance metrics that provide the objective measures to evaluate machine learning models for leukemia classification. Table 6 illustrates the performance metrics equations for two and multi-class leukemia classifications. In this table, TP, TN, FP, FN, and *C* indicate True Positive, True Negative, False Positive, False Negative, and the total number of classes, respectively. The accuracy is the number of samples that were correctly classified divided by the total number of samples. The actual number of accurately labeled positive images over the total number of positive images (correctly or incorrectly) is called precision. Sensitivity is all actual positive cases (leukemia), which are correctly identified, while specificity is all actual negative cases (healthy), which are correctly identified. F1-score provides a weighted average (harmonic mean) of precision and sensitivity. Figs 9 and 10 display the confusion matrix of the ResNet18 and MobileNetv4 models on the datasets with train-validation-test split in the test steps. We calculated the evaluation metrics using the obtained values of the confusion matrices for the ResNet18 and MobileNetv4 models and show them in Tables 7 and 8, respectively. The best values under different architectures are shown in bold. According to the obtained values in Tables 7 and 8, the ResNet18SCAB and MobileNetv4SCAB have achieved better performance metrics on all datasets than the other architectures. The ResNet18SCAB improves the accuracy of the ResNet18 by 2.02%, 2%, 3.1%, and 1.67% on the ALL, ALL-IDB2, C-NMC, and Mixture-Leukemia datasets, respectively, whereas these improvements have been 1.62%, 3%, 3.38%, and 1.18% by the MobileNetv4SCAB. The ResNet18SCAB increases the F1-score of the ResNet18 by 4.46%, 2.02%, 2.23%, and 2.52% on the ALL, ALL-IDB2, C-NMC, and Mixture-Leukemia datasets, respectively, whereas these increases have been 3.48%, 2.97%, 2.36%, and 2.28% by the MobileNetv4SCAB. Therefore, by comparing the obtained performance metrics of the models, we conclude that MobileNetv4SCAB is more efficient than the others.

**Table 6:** Performance metrics equations for leukemia classification.

Metric (%)	Equation (×100)	
	Two-class	Multi-class
Accuracy	$\frac{TP+TN}{TP+TN+FP+FN}$	$\frac{1}{C}\sum_{i=1}^{C}(\frac{(TP{)}_{i}+(TN{)}_{i}}{(TP{)}_{i}+(TN{)}_{i}+(FP{)}_{i}+(FN{)}_{i}})$
Precision	$\frac{TP}{TP+FP}$	$\frac{1}{C}\sum_{i=1}^{C}(\frac{(TP{)}_{i}}{\left(TP{)}_{i}+(FP{)}_{i}\right)})$
Sensitivity	$\frac{TP}{TP+FN}$	$\frac{1}{C}\sum_{i=1}^{C}(\frac{(TP{)}_{i}}{\left(TP{)}_{i}+(FN{)}_{i}\right)})$
Specificity	$\frac{TN}{TN+FP}$	$\frac{1}{C}\sum_{i=1}^{C}(\frac{(TN{)}_{i}}{\left(TN{)}_{i}+(FP{)}_{i}\right)})$
F1-score	$2\times (\frac{Precision\times Sensitivity}{Precision+Sensitivity})$	$\sum_{i=1}^{C}2\times \frac{Precision_{i}\times Sensitivity_{i}}{Precision_{i}+Sensitivity_{i}}$

**Table 7:** The performance metrics of the ResNet18 models.

Architecture	Dataset		Accuracy (%)	Precision (%)	Sensitivity (%)	Specificity (%)	F1-score (%)
ResNet18	ALL		97.39	94.44	94.44	98.53	94.31
ResNet18PCAB			98.67	97.22	97.24	99.22	97.2
ResNet18SCAB			99.41	98.76	98.78	99.65	98.77
ResNet18	ALL-IDB2		97	96	97.96	96.08	96.97
ResNet18PCAB			98	96	100	96.15	97.96
ResNet18SCAB			99	98	100	98.04	98.99
ResNet18	C-NMC	Fold-0	92.05	97.91	91.05	94.74	94.35
ResNet18PCAB			93.18	98.33	92.16	95.88	95.14
ResNet18SCAB			95.17	99.16	94.05	98	96.54
ResNet18		Fold-1	89.11	89.67	93.94	80.31	91.75
ResNet18PCAB			89.94	90.5	94.4	81.75	92.41
ResNet18SCAB			92.18	93.39	94.96	86.67	94.17
ResNet18		Fold-2	83.05	95.92	82.46	85.51	88.68
ResNet18PCAB			83.9	96.33	83.1	87.14	89.22
ResNet18SCAB			86.16	97.96	84.51	92.86	90.74
ResNet18		Average	88.07	94.5	89.15	86.85	91.59
ResNet18PCAB			89	95.05	89.89	88.26	92.26
ResNet18SCAB			91.17	96.84	91.17	92.51	93.82
ResNet18	Mixture-Leukemia		97.09	96.02	96.22	96.62	96.08
ResNet18PCAB			98.39	97.66	97.76	95.5	97.69
ResNet18SCAB			98.76	98.59	98.62	96.93	98.6

**Table 8:** The performance metrics of the MobileNetv4 models.

Architecture	Dataset		Accuracy (%)	Precision (%)	Sensitivity (%)	Specificity (%)	F1-score (%)
MobileNetv4	ALL		98.38	96.6	96.79	99.25	96.52
MobileNetv4PCAB			99.58	99.07	99.07	99.76	99.07
MobileNetv4SCAB			100	100	100	100	100
MobileNetv4	ALL-IDB2		97	98	96.08	97.96	97.03
MobileNetv4PCAB			99	100	98.04	100	99.01
MobileNetv4SCAB			100	100	100	100	100
MobileNetv4	C-NMC	Fold-0	93.75	99.58	91.89	98.92	95.58
MobileNetv4PCAB			95.45	99.58	94.07	98.99	96.75
MobileNetv4SCAB			97.16	99.58	96.36	99.05	97.94
MobileNetv4		Fold-1	91.34	91.32	95.67	83.46	93.45
MobileNetv4PCAB			92.74	93.39	95.76	86.89	94.56
MobileNetv4SCAB			94.69	95.87	96.27	91.45	96.07
MobileNetv4		Fold-2	85.59	99.18	83.22	96.77	90.5
MobileNetv4PCAB			87.01	99.18	84.67	97.01	91.35
MobileNetv4SCAB			88.98	99.59	86.52	98.61	92.6
MobileNetv4		Average	90.23	96.69	90.26	93.05	93.18
MobileNetv4PCAB			91.73	97.38	91.5	94.03	94.22
MobileNetv4SCAB			93.61	98.35	93.05	96.37	95.54
MobileNetv4	Mixture-Leukemia		98.22	96.96	97.16	94.8	97.02
MobileNetv4PCAB			99.06	98.59	98.7	96.99	98.62
MobileNetv4SCAB			99.4	99.3	99.3	97.77	99.3

Also, the accuracy for each fold and the average accuracy of the folds of the ResNet18 and MobileNetv4 models on the datasets with the 5-fold cross-validation split in the test step are calculated and shown in Tables 9 and 10, respectively. The values in brackets in these Tables indicate the standard deviation. The best values under different architectures are shown in bold. Based on the achieved values in Tables 9 and 10, the ResNet18SCAB and MobileNetv4SCAB have achieved higher accuracies on all datasets than the other architectures. The ResNet18SCAB enhanced the average accuracy of the ResNet18 by 1.86%, 2.24%, 2.04%, and 2.34% on the ALL, ALL-IDB2, C-NMC, and Mixture-Leukemia datasets, respectively, whereas these enhancements were 1.38%, 2.21%, 2.63%, and 2.12% by the MobileNetv4SCAB. Thus, by comparing the achieved average accuracies of the models, we conclude that MobileNetv4SCAB is more effective than the others.

**Table 9:** The accuracy of the ResNet18 models.

Architecture	Dataset	Accuracy (%)					
		Fold-0	Fold-1	Fold-2	Fold-3	Fold-4	Average
ResNet18	ALL	93.37	94.76	95.14	94.61	93.67	94.31 (±0.674)
ResNet18PCAB		93.89	94.92	96.08	95.42	94.26	94.91 (±0.786)
ResNet18SCAB		95.54	96.13	96.87	96.5	95.79	96.17 (±0.478)
ResNet18	ALL-IDB2	93.48	94.55	93.96	93.72	94.01	93.94 (±0.357)
ResNet18PCAB		95.21	95.38	94.7	95.11	95.19	95.12 (±0.227)
ResNet18SCAB		96.57	96.03	96.39	95.99	95.92	96.18 (±0.254)
ResNet18	C-NMC	84.88	85.73	84.63	86.69	84.41	85.27 (±0.840)
ResNet18PCAB		85.16	86.38	86.47	87.3	85.13	86.09 (±0.834)
ResNet18SCAB		86.34	86.91	87.58	88.53	87.17	87.31 (±0.732)
ResNet18	Mixture-Leukemia	94.97	92.81	92.24	94.06	90.79	92.97 (±1.449)
ResNet18PCAB		95.25	93.77	94.64	95.28	93.35	94.46 (±0.779)
ResNet18SCAB		95.83	95.2	95.45	95.95	94.41	95.31 (±0.549)

**Table 10:** The accuracy of the MobileNetv4 models.

Architecture	Dataset	Accuracy (%)					
		Fold-0	Fold-1	Fold-2	Fold-3	Fold-4	Average
MobileNetv4	ALL	96.42	95.86	97.61	96.21	97.03	96.63 (±0.622)
MobileNetv4PCAB		97.11	96.08	96.99	98.35	97.5	97.21 (±0.737)
MobileNetv4SCAB		97.31	97.87	98.26	98.43	98.18	98.01 (±0.394)
MobileNetv4	ALL-IDB2	94.87	96.54	95.76	96.93	94.71	95.76 (±0.880)
MobileNetv4PCAB		96.45	96.59	97.58	96.88	95.69	96.64 (±0.614)
MobileNetv4SCAB		98.42	97.2	97.61	98.05	98.55	97.97 (±0.504)
MobileNetv4	C-NMC	87.91	88.53	85.84	89.33	86.79	87.68 (±1.239)
MobileNetv4PCAB		89.5	87.92	87.71	88.56	88.65	88.47 (±0.629)
MobileNetv4SCAB		90.27	91.17	89.02	90.98	90.12	90.31 (±0.760)
MobileNetv4	Mixture-Leukemia	94.77	95.19	95.74	95.37	93.99	95.01 (±0.599)
MobileNetv4PCAB		96.38	95.92	96.28	96.14	95.81	96.11 (±0.214)
MobileNetv4SCAB		97.2	97.01	98.11	96.25	97.06	97.13 (±0.593)

### Computational complexities

Computational complexity is a measure of the amount of computing resources, such as time and space, that a specific machine learning model consumes when it runs. We calculated the number of parameters, model size, Floating-point operations per second (FLOPs), and Multiply-accumulate operations (MACs) of the Resnet18 and MobileNetv4 models and listed them in Table 11. The ‘M’, ‘MB’, and ‘G’ indicate Million, MegaByte, and GigaByte, respectively. The best accuracy values under different architectures are presented in bold. The ResNet18PCAB has 0.78 million parameters, 72.95 MegaBytes of model size, 140.31 GigaBytes of FLOPs, and 69.84 GigaBytes of MACs more than the ResNet18, while its accuracy is 1.3% more. The ResNet18SCAB increases 0.01 million parameters, 11.92 GigaBytes of model size, 0.17 GigaBytes of FLOPs, and 0.02 GigaBytes of MACs to the ResNet18, while improving the accuracy by 1.67%. Comparison of the number of parameters, model size, FLOPs, MACs, and accuracy of the ResNet18PCAB and ResNet18SCAB shows that ResNet18SCAB not only imposed lower computational complexities on the ResNet18 but also improved its accuracy more. The MobileNetv4PCAB imposes 23.52 million parameters, 213.81 MegaBytes of model size, 25.34 GigaBytes of FLOPs, and 12.65 GigaBytes of MACs to the MobileNetv4, while enhancing its accuracy is 0.84%. The MobileNetv4SCAB increases 0.32 million parameters, 9.27 MegaBytes of model size, 0.17 MegaBytes of FLOPs, and 0.05 GigaBytes of MACs to the MobileNetv4, while increasing the accuracy by 1.18%. Comparison of the number of parameters, model size, FLOPs, MACs, and accuracy of the MobileNetv4PCAB and MobileNetv4SCAB confirms that MobileNetv4SCAB not only imposed fewer computational complexities on the MobileNetv4 but also enhanced its accuracy more. Hence, we conclude that the SCAB is more efficient than the PCAB because it is a lighter and optimum block.

**Table 11:** Comparison of the time and computational complexities.

Architecture	Parameters (M)	Model size (MB)	FLOPs (G)	MACs (G)	Accuracy (%)
ResNet18	11.18	242.2	701.27	350.08	97.09
ResNet184PCAB	11.96	315.15	841.58	419.92	98.39
ResNet184SCAB	11.19	254.12	701.44	350.1	98.76
MobileNetv4	2.5	60.27	18.01	8.87	98.22
MobileNetv4PCAB	26.02	274.08	43.35	21.52	99.06
MobileNetv4SCAB	2.82	69.54	18.18	8.92	99.4

### Ablation study

An ablation study purposes to define the influence of each component on the efficiency of a machine learning model by replacing or eliminating it [48]. We performed two experiments to clarify the individual contributions of the type of pooling and convolutional layers within the proposed CABs in a comprehensive ablation study. In the first set of experiments, the impact of various pooling layers, including GAP, GMP, and joint GAP and GMP, on the enhancement of the network performance was compared, and the results of these experiments are demonstrated in Table 12. The best values are shown in bold. It is evident from Table 12 that the performance metrics of the MobileNetv4 are increased by combining GAP and GMP. They assist in preserving key features and making the model more robust to small variations in the input.

**Table 12:** Ablation experiment data for different pooling layers.

Architecture	Accuracy (%)	Precision (%)	Sensitivity (%)	Specificity (%)	F1-score (%)
MobileNetv4	98.22	96.96	97.16	94.8	97.02
MobileNetv4PCAB (GAP)	98.86	98.36	98.44	96.29	98.38
MobileNetv4PCAB (GMP)	98.54	97.66	97.8	95.54	97.7
MobileNetv4PCAB (GAP + GMP)	99.06	98.59	98.7	96.99	98.62
MobileNetv4SCAB (GAP)	99.17	99.06	99.11	97.05	99.07
MobileNetv4SCAB (GMP)	98.86	98.36	98.46	96.29	98.39
MobileNetv4SCAB (GAP + GMP)	99.4	99.3	99.3	97.77	99.3

In the second set of experiments, the roles of standard and dilation convolution layers are investigated. Table 13 presents the results of these experiments that were performed using different convolution layers. The best values are indicated in bold. It can be confirmed that the DC layer outperforms standard convolution. The DC layer can capture features at multiple scales by an escalated kernel size without increasing parameters. Also, it reduces spatial resolution loss compared to standard convolutions with larger filters.

**Table 13:** Ablation experiment data for different convolution layers.

Architecture	Accuracy (%)	Precision (%)	Sensitivity (%)	Specificity (%)	F1-score (%)
MobileNetv4	98.22	96.96	97.16	94.8	97.02
MobileNetv4PCAB (Standard)	98.65	98.13	98.28	95.6	98.16
MobileNetv4PCAB (Dilation)	99.06	98.59	98.7	96.99	98.62
MobileNetv4SCAB (Standard)	98.55	97.89	98.47	94.99	98.38
MobileNetv4SCAB (Dilation)	99.4	99.3	99.3	97.77	99.3

### Comparison with existing models

We compare the performance metrics of the proposed model, such as accuracy, precision, sensitivity, specificity, and F1-score, with previous state-of-the-art models on the ALL, ALL-IDB2, C-NMC, and Mixture-Leukemia datasets, and they are presented in Table 14. The best values are shown in bold. As we can see, the proposed model has achieved higher performance criteria values than prior existing models on all datasets.

**Table 14:** Overall comparison of the model with existing models.

Study	Approach	Year	Dataset	Accuracy (%)	Precision (%)	Sensitivity (%)	Specifity (%)	F1-score (%)
Praveena and Singh [32]	Hybrid	2020	ALL-IDB2	93.5	-	95.28	93.89	-
Sahlol et al. [33]			ALL-IDB2	96.11	-	93	95	-
			C-NMC	83.3	-	91.1	67.3	-
Das and Meher [34]	Deep learning	2021	ALL-IDB2	97.18	98.52	95.9	98.46	97.19
Ghaderzadeh et al. [21]			ALL	99.85	99.74	99.52	99.89	99.63
Zakir Ullah et al. [28]			C-NMC	91.1	89.45	92.32	90.25	90.7
Jawahar et al. [35]		2022	C-NMC	91.13	93	98	-	96
Masoudi [29]	Hybrid		ALL-IDB2	99.6	99.67	99.4	-	99.5
Abhishek et al. [36]		2023	Mixture	84	-	-	-	-
Atteia et al. [37]			ALL	97.4	-	-	-	-
Rahman et al. [38]			ALL	99.84	99.75	99.87	-	99.81
Awais et al. [39]		2024	ALL	98.69	98.71	98.72	99.57	98.71
			ALL-IDB2	99.15	99.44	98.88	99.15	99.44
Jawahar et al. [30]	Deep learning		ALL	91.98	-	-	-	-
Shah et al. [40]	Machine learning		C-NMC	92.5	-	-	-	94.6
Kasim et al. [41]	Hybrid	2025	Mixture	88	86	87	-	87
Nagendiran and Murugasamy [42]	Machine learning		ALL	98.23	-	-	-	98.23
			ALL-IDB2	98.07	-	-	-	98.07
Shaban [43]			ALL	99.2	90.5	89	89.9	89.7
Proposed Method	Deep learning		ALL	**100**	**100**	**100**	**100**	**100**
			ALL-IDB2	100	100	100	100	100
			C-NMC	93.61	98.35	93.05	96.37	95.54
			Mixture	99.4	99.3	99.3	97.77	99.3

### Statistical analysis

**Fig 11:**
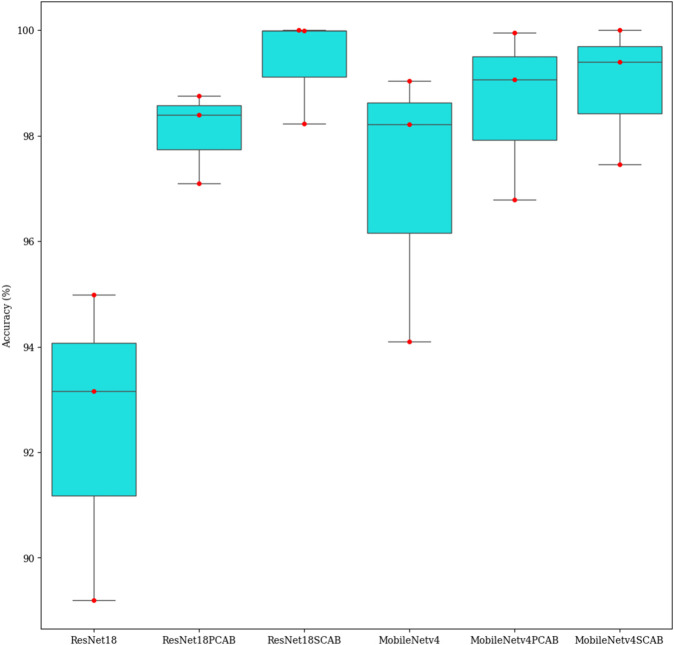
The ANOVA results for models.

Analysis of Variance (ANOVA) is a statistical analysis method that is usually used to study differences between the averages of two or more groups. In machine learning, the ANOVA test helps us to measure which components or features are more important [49]. In this paper, the PCAB and SCAB are the components that we are trying to understand which one has more effect on the improvement of the network’s performance. Fig 11 presents the graphical examination of the ANOVA test for the results of the accuracy of the models. According to this figure, the difference in average accuracy between the ResNet18 and MobileNetv4 models is quite obvious. The attention-based models have also achieved higher accuracies than others. We can see that the SCAB increased the classification accuracy of the models more than the PCAB.

### Visualizing feature maps

**Fig 12:**
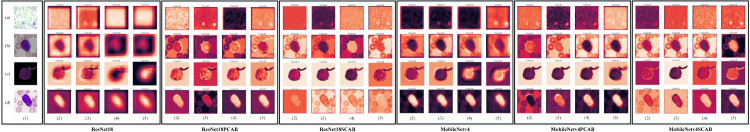
Feature maps from the last convolution layers of the models on the datasets. (a∼d) are the images and their feature maps from the ALL, ALL-IDB2, C-NMC, and Mixture-Leukemia datasets, whereas (1) include input images, (2∼5) are the output feature maps of the last convolution layers of each model.

Understanding and interpreting the behavior of each deep-learning model is easier by visualizing feature maps. It helps to know what features are being detected at different layers of the network, which is vital for developing insight into its inner workings and optimizing its architecture. We randomly chose an image from each dataset and visualized its feature maps from the last convolution layers of the networks. Fig 12 displays the input images and their feature maps from the last convolution layers of the models on the datasets. The attention-based models extracted the feature maps that separate the border of WBCs from their backgrounds clearly. This separation is performed better by the models with SCAB than the models with PCAB. Thus, we conclude that the channel attention sub-block of the SCAB contributes more to baseline models for extracting the features related to the shape and texture of WBCs than the PCAB.

**Fig 13:**
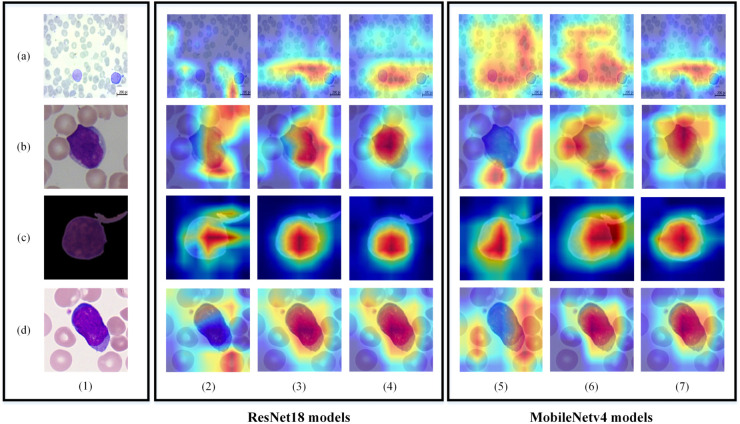
Grad-CAM images from the last convolution layers of the models on the datasets. (a∼d) are the images and their activation maps from the ALL, ALL-IDB2, C-NMC, and Mixture-Leukemia datasets, whereas (1) include input images, (2∼7) are the Grad-CAM images extracted from the last convolution layers of the ResNet18, ResNet18PCAB, ResNet18SCAB, MobileNetv4, MobileNetv4PCAB, and MobileNetv4SCAB, respectively.

The Gradient-weighted Class Activation Mapping (Grad-CAM) is a technique that finds out which parts of an input image have led a deep-learning model to its final decision. It involves making heat maps representing the activation classes on the input images [50]. An input image from each training set is accidentally selected, and their Grad-CAM from the last convolution layers of the models on the datasets are visualized and displayed in Fig 13. As we can see, the heat maps of the attention-based models cover a larger area of the WBCs. The heat maps of the models based on SCAB are more precise and focused on the whole regions of the WBCs than others. Therefore, we conclude that the spatial attention sub-block of the SCAB helps more to the models for extracting the location of the WBCs than the PCAB.

#### The cases of correct and incorrect predictions.

**Fig 14:**
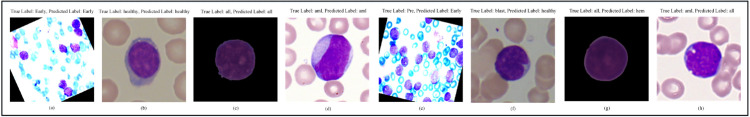
Samples of images of correct and incorrect prediction of the datasets. (a∼d) are correctly classified while (e∼h) are misclassified.

The proposed model was able to correctly identify the class of many samples in the dataset, while the class of some samples was misclassified. Fig 14 shows samples of images of correct and incorrect predictions of the datasets. The structure and location of the samples are well extracted by the proposed model, but it made a mistake in predicting some of the classes of WBCs. For example, the true classes of the (f) and (g) images were unhealthy, whereas their circular structure is preserved, and they are quite similar to healthy cells. Such similarities even cause doctors to make mistakes in correctly diagnosing the class of such samples. Therefore, the existence of the similarity of the WBCs in different classes has been one of the main reasons for the networks’ error in predicting their classes correctly.

## Conclusion and future work

Distinguishing normal cells from immature leukemia blasts under the microscope is hard in the manual leukemia classification method because they have almost identical appearances in terms of morphology. Two attention-based CNNs using human cognitive attention mechanisms are designed and developed for the automatic classification of leukemia from microscopic images in this study. They force the CNNs to extract the robust features related to the structure and location of the WBCs from microscopic images. Two cognitive attention blocks, the PCAB and SCAB, are embedded in the architecture of the ResNet18 and MobileNetv4 to generate the four cognitive attention-based networks. The ResNet18, ResNet18PCAB, ResNet18SCAB, MobileNetv4, MobileNetv4PCAB, and MobileNetv4SCAB are implemented on the ALL, ALL-IDB2, C-NMC, and Mixture-Leukemia datasets with the same hyperparameters, hardware, and software conditions. The comprehensive comparison between the obtained results of the models confirmed that the MobileNetv4SCAB is more efficient for leukemia classification than the others and the previously existing models. Hence, we introduce it as the proposed model in this paper. The proposed blocks can be improved and embedded in the architecture of the other state-of-the-art CNNs to produce optimized attention-based models for future work. Also, the proposed method can be enhanced for execution on other similar datasets.
